# Students’ performance in the different clinical skills assessed in OSCE: what does it reveal?

**DOI:** 10.3402/meo.v20.26185

**Published:** 2015-02-18

**Authors:** Joong Hiong Sim, Yang Faridah Abdul Aziz, Azura Mansor, Anushya Vijayananthan, Chan Choong Foong, Jamuna Vadivelu

**Affiliations:** 1 Medical Education & Research Development Unit, Faculty of Medicine, University of Malaya, Malaysia; 2 Department of Biomedical Imaging, Faculty of Medicine, University of Malaya, Malaysia; 3 Department of Orthopaedic Surgery, Faculty of Medicine, University of Malaya, Malaysia

**Keywords:** OSCE, clinical skills, student performance

## Abstract

**Introduction:**

The purpose of this study was to compare students’ performance in the different clinical skills (CSs) assessed in the objective structured clinical examination.

**Methods:**

Data for this study were obtained from final year medical students’ exit examination (*n*=185). Retrospective analysis of data was conducted using SPSS. Means for the six CSs assessed across the 16 stations were computed and compared.

**Results:**

Means for history taking, physical examination, communication skills, clinical reasoning skills (CRSs), procedural skills (PSs), and professionalism were 6.25±1.29, 6.39±1.36, 6.34±0.98, 5.86±0.99, 6.59±1.08, and 6.28±1.02, respectively. Repeated measures ANOVA showed there was a significant difference in the means of the six CSs assessed [*F*(2.980, 548.332)=20.253, *p*<0.001]. Pairwise multiple comparisons revealed significant differences between the means of the eight pairs of CSs assessed, at *p*<0.05.

**Conclusions:**

CRSs appeared to be the weakest while PSs were the strongest, among the six CSs assessed. Students’ unsatisfactory performance in CRS needs to be addressed as CRS is one of the core competencies in medical education and a critical skill to be acquired by medical students before entering the workplace. Despite its challenges, students must learn the skills of clinical reasoning, while clinical teachers should facilitate the clinical reasoning process and guide students’ clinical reasoning development.

In medical education, the use of objective structured clinical examination (OSCE) in the assessment of clinical competence has become widespread since it was first described by Harden and Gleeson (1).

Although the OSCE assesses clinical skills (CSs), the concept of ‘CS’ is not clearly defined in the literature. CS seems to mean different things to different people, with a lack of clarity as to what is, and what is not a CS (2). Different authors (2–5) include different domains within their definitions of CS. Michels and colleagues (2) include physical examination skills, communication skills, practical skills, treatment skills, and clinical reasoning or diagnostic skills as CSs. However, Junger and colleagues (3) refer to CS as physical examination skills only whilst Kurtz and colleagues (4) also consider communication skills as a CS. The Institute for International Medical Education (5) adopts a broader perspective in which history taking, physical examination, practical skills, interpretation of results, and patient management come under the headings of CSs.

According to Michels and colleagues (2), acquiring CSs involves learning how to perform the skills (procedural knowledge), the rationale for doing (underlying basic sciences knowledge), and interpretation of the findings (clinical reasoning). Without these three components, CS is merely a mechanical performance with limited clinical application. However, clinicians are often unaware of the complex interplay between different components of a CS that they are practicing and consequently do not teach all these aspects to students (2).

One of the core competencies in medical education is clinical reasoning (6). Clinical reasoning plays a major role in the ability of doctors to make a diagnosis and reach treatment decisions. According to Rencic (7), clinical reasoning is one of the most critical skills to teach to medical students. Case presentation is frequently used in most clinical teaching settings and although the key role of clinical teachers is to facilitate and evaluate case presentations and give suggestions for improvement (8), clinical teachers rarely have adequate training on how to teach clinical reasoning skills (CRSs) (7). Consequently, learners often receive only vague coaching on the clinical reasoning process (9).

Clinical reasoning has been a topic of research for several decades, yet there still exists no clear consensus regarding what clinical reasoning entails and how it might best be taught and assessed (10). Durning and colleagues (10) found this lack of consensus could be due to contrasting epistemological views of clinical reasoning and the different theoretical frameworks held by medical educators.

This study adopted Miller's pyramid of clinical competence (11) as the conceptual basis for the assessment of CSs. Miller's pyramid outlines the issues involved when analysing validity. The pyramid conceptualises the essential facets of clinical competence. It proposes clinical competence in multiple levels: ‘knows’, ‘knows how’, ‘shows how’, and ‘does’ (Fig. 1). A candidate ‘knows’ first before progressing to ‘knows how’. ‘Knows’ is analogous to factual knowledge whereas ‘knows how’ is equivalent to concept building and understanding. At a higher level, a candidate ‘shows how’, that is, develops the competence to ‘perform’. At the highest level, the candidate ‘does’, that is, actually carries out the tasks competently in real-life situations.

**Fig. 1:**
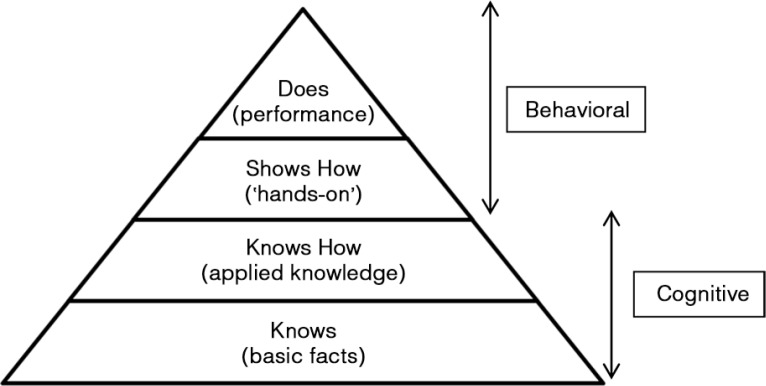
Miller's pyramid of clinical competence. Source: Adapted from Ref. (11).

Although there are numerous studies on OSCEs, most of these studies focused on issues of validity, reliability, objectivity, and standard setting of OSCEs (12–16). In this study, we sought to examine and compare students’ performance in the six CSs assessed in the OSCE. Instead of interpreting OSCE stations as a whole, this investigation would reveal students’ specific strengths and weaknesses in the different CSs assessed. This study attempted to answer the research question: ‘Is there any significant difference in students’ performance among the six CSs assessed in the OSCE?’

In the context of this study, the six CSs were history taking, physical examination, communication skills, CRS, procedural skills (PSs), and professionalism. These six skills were included in the OSCE as they were considered by the authors’ institutional OSCE and curriculum committee as core competencies to be attained as a reasonable requirement at graduation for the degrees of Bachelor of Medicine and Bachelor of Surgery (MBBS) candidates in the Malaysian context.

## Methods

This is a retrospective study analysing secondary data. The study population comprised 185 final year medical students who took the OSCE in their exit examination.

### Institutional setting

MBBS is a 5-year programme. The programme is divided into three phases: Phase 1 (1 yr), Phase 2 (1 yr), and Phase 3 (3 yrs). Phase 3 (clinical years) is further divided into Phase 3A and Phase 3B of 1½ yrs each. During each phase of study, course assessment consists of continuous assessment and professional examinations. Phase 3B students take the final MBBS Examination that comprises four components: Component A (end-of-posting tests), Component B (two theory papers), Component C (one long case and three short cases), and Component D (OSCE).

### The OSCE

The OSCE comprised 16 work stations and 1 rest station. A time of 5 min was allocated for each station, with a 1-min gap between stations. Hence, each OSCE session took approximately 100 min. The examination was run in three parallel circuits of 17 stations each and was conducted over four rounds from morning until late afternoon. For the 16-station OSCE, nine were interactive and seven were non-interactive. Table 1 provides a summary of the OSCE stations.

**Table 1:** The OSCE stations

				Station score			Clinical skill assessed					
					
No.	Station objective	Department	Station type	Mean	Min	Max	History taking	Physical exam	Comm. skills	CR skills	Proc. skills	Prof
S01	Rest station	–	Rest	–	–	–	–	–	–	–	–	–
S02	To assess candidates’ ability to calculate anion gap and interpret ABG	EME	NA	5.41	0.0	8.0				√		
S03	To assess candidate's skills in performing a visual field examination	OPH	IA	7.60	0.0	10.0		√	√		√	√
S04	To assess candidate's ability to treat a dehydrated paediatric patient	PED	NA	4.87	0.0	10.0				√		
S05	To assess candidate's knowledge of common drugs (magnesium sulphate)	OBG	NA	6.61	0.0	9.5				√		
S06	To assess candidate's ability to interpret radiological findings and communicate this to the patient	RAD	IA	5.73	0.0	10.0			√	√		√
S07	To assess candidate's ability to plan for the transport of the critically ill patient	ANE	NA	6.99	0.0	9.5				√		
S08	To assess candidate's ability to diagnose and treat a common nasal illness	ENT	NA	5.43	0.0	10.0				√		
S09	To assess candidate's ability to perform CPR	EME	IA	7.00	0.0	10.0			√	√	√	√
S10	To assess candidate's communication skills in explaining a common problem (prolapsed disc) to the patient	ORS	IA	5.19	0.0	9.0		√	√	√		√
S11	To assess candidate's ability to perform and interpret Rinne's test	ENT	IA	7.69	0.0	10.0			√	√	√	√
S12	To assess candidate's communication skills on ability to counsel patient on dietary advice	PCM	IA	5.60	0.0	10.0	√		√			√
S13	To assess candidate's ability to elicit first rank symptoms in Schizoprenia	PSY	IA	6.91	0.0	10.0	√		√	√		√
S14	To assess candidate's ability to teach the patient how to use an inhaler	PCM	IA	5.27	0.0	10.0			√		√	√
S15	To assess candidate's awareness of hypothermia in the operation theatre	ANE	NA	5.92	0.0	10.0					√	
S16	To demonstrate the ability to clinically assess a patient with post-operative fever	SUR	IA	6.08	0.0	10.0			√	√	√	
S17	To assess candidate's ability to interpret fundoscopy findings	OPH	NA	3.60	0.0	8.0				√		

EME=Trauma & Emergency; OPH=Opthalmology; PED=Paediatrics; OBG=Obstetrics & Gynaecology; RAD=Radiology; SUR=Surgery; ANE=Anaesthetics; ENT=Otolaryngology; ORS=Orthopaedic Surgery; PCM=Primary Care Medicine; PSY=Psychological Medicine; IA=Interactive; NA=Non-Interactive; Comm=Communication; CR=Clinical Reasoning; Proc=Procedural; Prof=Professionalism

Each station's score sheet contained a detailed checklist of items examined (total=10 marks). A global rating was also included for the examiner to indicate the global assessment for the station. For interactive stations, both checklists and global ratings were used for scoring. For non-interactive stations that involved data interpretation, no examiner was present and only checklists were used.

### Validity and reliability of the OSCE

Various measures were taken to ensure a high validity and reliability for the OSCE.

Content validity was determined by how well the test content mapped across the learning objectives of the course (17). Content validity of the OSCE was established by blueprinting. This ensured adequate sampling across subject areas and skills, in terms of the number of stations covering each skill and the spread over the content of the course being tested. For quality assurance of the OSCE stations, question vetting was conducted at both the department and the faculty level.

Station materials were written well in advance of the examination date. For each station, there were clear instructions for the candidates and notes for the examiners, list of equipment required, personnel requirements, scenario for standardised patients, and marking schedule. The stations were reviewed and field-tested prior to the actual examination.

Consistency of marking among examiners contributes to reliability. Consistent performance of standardised patients ensures each candidate is presented with the same challenge. To ensure consistency and fairness of scores, training of examiners and standardised patients was conducted by the Faculty of Medicine OSCE Team. To further enhance reliability, structured marking schedules allowed for more consistent scoring by examiners according to predetermined criteria on the checklists.

To increase reliability, it is better to have more stations with one examiner per station than fewer stations with two examiners per station (18). As candidates have to perform a number of different tasks across different stations, this wider sampling across different cases and skills results in a more reliable picture of a candidate's overall competence. Furthermore, as the candidates move through all the stations, each is examined by a number of different examiners. Multiple independent observations are collated while individual examiner bias is attenuated.

In this study, the 16 OSCE stations from 11 clinical departments allowed wider sampling of content. Furthermore, most of the stations assessed multiple skills (Table 1). Hence, both the validity and reliability of the examination were enhanced.

### Data collection and data analysis

After obtaining approval to perform the study from the Faculty of Medicine, University of Malaya, raw scores for each of the 16 stations for all the 185 candidates were obtained from examination section, Faculty of Medicine. Subsequently, retrospective analysis of data was conducted using IBM SPSS version 22. An alpha level of 0.05 was set for all the statistical tests.

### Statistical analysis

To check for internal consistency of the OSCE, Cronbach's alpha was computed across the 16 stations for all the candidates (*n*=185).

Since each OSCE station assessed one or more skills (Table 1), mean scores for each of the six CSs assessed across the 16 stations were also computed. For example, to compute the mean score for PS, the mean score for stations S03, S09, S11, S14, S15, and S16 would be computed. Hence, mean score for PS=(7.60+7.00+7.69+5.27+5.92+6.08)/6=6.59.

Because the 185 candidates constituted a single group, means for the six different CSs assessed were compared using repeated measures ANOVA or within-subjects ANOVA (19). Repeated measures ANOVA is the equivalent of the one-way ANOVA but for related, not independent groups, and is the extension of the paired sample *t*-test (19–21). In this study, the independent variable or factor was CSs with six levels (history taking, physical examination, communication skills, CRS, PS, and professionalism). The dependent variable was the mean score for each CS assessed.

The design of repeated measures ANOVA is based on the assumption of sphericity, which is equivalent to Levene's test for equality of variance in one-way ANOVA. The statistic used in repeated measures ANOVA is *F*, the same statistic as in simple ANOVA. If the *F* value is significant, *post-hoc* tests were conducted using pairwise multiple comparisons with Bonferonni correction for type I error (19).

In this study, a repeated measures design was used because 1) in using the same subject, a repeated measure allows the researcher to exclude the effects of individual differences that could occur in independent groups (22), 2) the sample size is not divided between groups and thus inferential testing becomes more powerful, 3) this design is economical when sample members are difficult to recruit because each member is measured under all conditions (22), and 4) the results can be directly compared, which can be problematic for independent measures (22).

## Results

Reliability analysis reported an alpha value of 0.68. This indicated the OSCE had moderate reliability.

Mean scores for all six CSs fell below seven out of a total possible score of ten, indicating the CSs of these students (*n*=185) were just at the satisfactory level. Means and standard deviations for history taking, physical examination, communication skills, CRS, PS, and professionalism were 6.25±1.29, 6.39±1.36, 6.34±0.98, 5.86±0.99, 6.59±1.08, and 6.28±1.02, respectively. Mean for PSs was the highest while mean for CRS was the lowest, among the six CSs assessed.

Results of the repeated measures ANOVA are shown in Tables 2–4. Table 2 shows the results of Mauchly's test of sphericity. Table 3 displays the results of the main ANOVA test and Table 4 summarises the results of multiple comparisons between each pair of CS assessed.

**Table 2:** Mauchly's Test of sphericity^a^

					Epsilon^b^		
					
Within subject effects	Mauchly's W	Approx. Chi-Square	df	Sig.	Greenhouse-Geisser	Huynh-Feldt	Lower-bound
Clinical skills assessed	0.050	546.824	14	**0.000**	**0.596**	0.607	0.200

a Design: Intercept

Within Subjects Design: Clinical skills assessed

b May be used to adjust the degrees of freedom for the average tests of significance. Corrected tests are displayed in Table 3

**Table 3:** Tests of within-subjects effects

Sources of variation	Sum of squares	df	Mean square	*F*	Sig.
Clinical skills					
Sphericity assumed	51.876	5	10.375	20.253	0.000
**Greenhouse-Geisser**	51.876	**2.980**	17.408	**20.253**	**0.000**
Huynh-Feldt	51.876	3.035	17.095	20.253	0.000
Lower-bound	51.876	1.000	51.876	20.253	0.000
Error (clinical skills)					
Sphericity assumed	471.283	920	0.512		
**Greenhouse-Geisser**	471.283	**548.332**	0.859		
Huynh-Feldt	471.283	558.355	0.844		
Lower-bound	471.283	184.000	2.561		

**Table 4:** Multiple comparisons (pair wise) of mean scores for the clinical skills assessed

Pairs of skills compared	Mean difference	Std. error	Sig.^a^
History–examination	−0.143	0.112	1.000
History–communications	−0.087	0.074	1.000
History–CRS	0.380*	0.091	0.001
History–procedural skills	−0.340*	0.096	0.008
History–professionalism	−0.031	0.072	1.000
Examination–communication	0.055	0.077	1.000
Examination–CRS	0.523*	0.089	0.000
Examination–procedural skills	−0.197	0.089	0.425
Examination–professionalism	0.112	0.075	1.000
Communication–CRS	0.467*	0.049	0.000
Communication–procedural skills	−0.252*	0.039	0.000
Communication–professionalism	0.056	0.027	0.531
CRS–procedural skills	−0.719*	0.062	0.000
CRS–professionalism	−0.411*	0.061	0.000
Procedural skills–professionalism	0.309*	0.054	0.000

a Adjustment for multiple comparisons: Bonferroni correction

* Mean difference is significant at the 0.05 level

CRS=Clinical Reasoning Score

Mauchly's test of sphericity is significant [*x* ^*2*^(14)=546.824, *p*<0.001]. This indicates that the assumption of sphericity had been violated (Table 2). Hence, adjustment of the degrees of freedom (df) for the test of within-subjects effects needs to be done. Because Epsilon<0.75, the Greenhouse–Geisser correction factor of 0.596 was used to compute the new df (23). New df1=2.980, new df2=548.332 (Table 3).

Based on the new df, repeated measures ANOVA still show a significant difference in the mean scores of the six CSs assessed [*F*(2.980, 548.332)=20.253, *p*<0.001] (Table 3).

*Post-hoc* tests were subsequently conducted to determine which pairs of means were significantly different. Pairwise multiple comparisons with Bonferroni correction revealed significant differences between the mean of eight pairs of CSs assessed, at *p*<0.05 (Table 4).

## Discussion

From the findings of the study, it could be concluded that students were weakest in their CRS. CRS (mean=5.86) is probably the most demanding compared to the other five CSs assessed (24–27). Clinical reasoning is a complex process that requires cognition and metacognition, as well as case and content-specific knowledge to gather and analyse patient's information, evaluate the significance of the findings, and consider alternative actions (24). According to Dennis (25), CRS may in some cases require the student to integrate findings from the enquiry plan (which involves history taking, physical examination, and investigations) into a clinical hypothesis and consider these findings in the overall context of the patient. This may include an ability to integrate history and physical examination to develop an accurate and comprehensive problem list, as well as a logical list of differential diagnoses (Station S08); an ability to interpret clinical data (Stations S02, S06, and S17); to recognise common emergency situations and demonstrate knowledge of an appropriate response (Station S04); and patient management (Station S07) (Table 1). Qiao and colleagues (26) found that clinical reasoning is a high-level, complex cognitive skill. Because clinical reasoning involves synthesising details of patient information into concise yet accurate clinical assessment, it is a higher order thinking skill (27). According to Krathwohl (27), synthesising is the highest cognitive level in the cognitive domain of the revised Bloom's taxonomy. Given the fact that each station was only allocated a time of 5 min, CRS could be a high cognitive resource activity for the candidates in a high-stakes exam, for instance, an OSCE in the exit examination.

In this study, the low mean score for CRS could be due to candidates’ inadequate knowledge of basic and clinical sciences (BCS) or the inability to apply this knowledge appropriately and reflectively in a clinical setting, or both. The assumption is made that clinical reasoning depends heavily on a relevant knowledge base. According to Miller's pyramid of clinical competence (Fig. 1) (11, 16), an OSCE lies in the level of ‘show how’, which is mainly behavioural (11). To perform at the level of ‘show how’ (behavioural), students need to have a strong knowledge base at the levels of ‘know’ and ‘know how’ (cognitive). Therefore, knowledge of BCS must be available and needs to be activated and retrieved before the student is able to perform or demonstrate the skill to ‘show how’. Students need to be able to apply their knowledge of BCS to help them better understand patients’ problems. Further study is needed to collect empirical data to verify these assumptions.

A longitudinal study at the authors’ institution revealed that students in the clinical years encountered challenges in recalling their knowledge of BCS that had been learned in preclinical years (28). The previous study echoes findings of this study in which the curriculum in the clinical years should provide more opportunities for students to revisit their knowledge of BCS learned during preclinical years. Information Process Theory (29) suggests learners need to retrieve and rehearse their learned knowledge regularly to retain the knowledge in their long-term memory. Formal lectures could be an appropriate platform for rehearsal (30) in addition to existing modes of teaching whereby students clerk patients, write case summaries, and present cases. Meanwhile, the acquisition of CRS primarily results from dealing with multiple patients over a period of time. Such patient–doctor interaction facilitates the availability and retrieval of conceptual knowledge through repetitive, deliberate practice (31). Exposure to multiple cases is crucial as clinical reasoning is not a generic skill. It is case or content specific (32). In the context of the authors’ institution, students are mainly observers who are neither directly involved in actual diagnosis of real patients nor explicitly required to practise CRS to fulfil the logbook requirements. Hence, it is up to the clinical teachers on how to teach CRS. It is suggested that during clerkships, ward rounds, or bedside teaching, clinical teachers should emphasise the clinical reasoning processes that show how a clinician arrives at a particular diagnostic or treatment decision to help students develop an understanding of how clinical decisions are made (25). More emphasis should be placed on diagnosis and management rather than basic mechanisms to prepare the students for the workplace. In addition, students should be given more hands-on experience of the clinical reasoning process, under the guidance and supervision of their clinical teachers. Although Gigante (9) pointed out that deliberate teaching of clinical reasoning may appear overwhelming and at times impossible, he explored how the clinical reasoning process can be taught in a stepwise fashion to students. Fleming and colleagues (33) used the concepts of problem representation, semantic qualifiers, and illness scripts to show how clinical teachers can guide students’ clinical reasoning development. Nonetheless, these authors cautioned that although clinical teachers can maximise students’ clinical exposure and experience, they cannot build illness scripts for them. Students must construct their own illness scripts based on real patients they have seen.

Several limitations of this study should be considered. The 5-min duration of each OSCE station may be relatively short compared to other medical schools. Data obtained from only one cohort of final year medical students from a single institution may limit generalisability of the findings. Hence, findings of this study need to be interpreted with caution when applied to other institutional settings.

## Conclusions

The final year of undergraduate medical education is crucial in transforming medical students into competent and reflective practitioners. Students’ unsatisfactory performance in CRS needs to be addressed as it is a core competency in medical education and a critical skill to be acquired by medical students before entering the workplace as health care practitioners. Despite its challenges, students must learn the skills of clinical reasoning for better patient care. Clinical teachers should facilitate the clinical reasoning process and guide students’ clinical reasoning development. Relying on time and experience to develop these skills is inadequate.

As research to uncover students’ educational needs for learning clinical reasoning during clerkships is limited (34), it is an area to explore in future studies.
